# Bias sputtering of granular L1_0_-FePt films with hexagonal boron nitride grain boundaries

**DOI:** 10.1038/s41598-023-38106-9

**Published:** 2023-07-08

**Authors:** Chengchao Xu, B. S. D. Ch. S. Varaprasad, David E. Laughlin, Jian-Gang Zhu

**Affiliations:** 1 Data Storage Systems Center, Carnegie Mellon University, Pittsburgh, PA 15213 USA; 2 Electrical and Computer Engineering Department, Carnegie Mellon University, Pittsburgh, PA 15213 USA; 3 Materials Science and Engineering Department, Carnegie Mellon University, Pittsburgh, PA 15213 USA

**Keywords:** Magnetic properties and materials, Information storage, Nanoparticles

## Abstract

In this paper, we present an experimental study of L1_0_-FePt granular films with crystalline boron nitride (BN) grain boundary materials for heat assisted magnetic recording (HAMR). It is found that application of a RF substrate bias (*V*_DC_ = -15 V) yields the formation of hexagonal boron nitride (*h*-BN) nanosheets in grain boundaries, facilitating the columnar growth of FePt grains during sputtering at high temperatures. The *h*-BN monolayers conform to the side surfaces of columnar FePt grains, completely encircling individual FePt grains. The resulting core–shell FePt-(*h*-BN) nanostructures appear to be highly promising for HAMR application. The high thermal stability of *h*-BN grain boundaries allows the deposition temperature to be as high as 650℃ such that high order parameters of FePt L1_0_ phase have been obtained. For the fabricated FePt-(*h*-BN) thin film, excellent granular microstructure with FePt grains of 6.5 nm in diameter and 11.5 nm in height has been achieved along with good magnetic hysteresis properties.

## Introduction

For heat-assisted magnetic recording (HAMR), L1_0_-FePt granular thin films have been the choice of recording media mainly due to the material’s ultra-high magneto-crystalline anisotropy at room temperature and the anisotropy-temperature dependence characteristics near the Curie point. Over the past two decades, extensive research and development efforts have been devoted to improving the microstructure with relatively thermal-insulating grain boundary materials (GBM) to fabricate columnar-shaped tall (height > 10 nm), small (diameter < 8 nm) and L1_0_-ordered FePt grains^1,2^. The formation of the L1_0_ phase of FePt requires elevated substrate temperatures during deposition, which poses a great challenge in achieving the desirable microstructure. Searching for an ideal GBM that enables the desired microstructure at a relatively high deposition temperature, commonly above 600℃, has been the constant focus of research in this area.

A list of GBMs, particularly carbon^3–6^, various oxides^7–14^, and boron nitride^15–17^ have been given a great deal of attention over the past. Although amorphous carbon as the GBM enables well-separated grains, it fails to promote columnar growth^4,5^. The spherical shape of the FePt grains in the FePt-C film limits the grain height to be similar to the in-plane grain diameter. Attempts to produce taller grains often lead to the formation of second-layer FePt grains that are incorrectly oriented and poorly ordered. Certain amorphous oxides like TiO_2_^7,8^, SiO_2_^9,11,12^, and TaO_x_^13^ have shown promise in promoting columnar growth; however, their low thermal stability (low melting point) has prevented them from being adopted. For example, silicon oxide typically enables columnar growth but often fails to encircle FePt grains at high temperature, resulting in many lateral connections between neighboring grains^11^. It has been suggested that the actual melting temperature of the sputtered SiO_2_ GBM might be substantially lower than that of the bulk form^12^. In order to inhibit the lateral growth of FePt grains at process temperatures higher than 600℃, a GBM with higher thermal stability is required.

It has been shown that amorphous boron nitride (*a*-BN) as a GBM holds some advantages over carbon and SiO_2_. However, when the FePt-(*a*-BN) film thickness exceeds 4 nm, it fails to provide good grain separation^15^. Several techniques have been attempted to improve both the microstructure and chemical ordering of FePt-(*a*-BN) film, including adding N_2_ to the sputtering atmosphere^18^, mixing with other GBM^19,20^, and applying DC substrate bias^21^. However, none of these approaches have yielded promising outcomes regarding the columnar growth of FePt grains.

Another alternative is to replace *a*-BN with crystalline BN for the grain boundaries. The crystalline hexagonal boron nitride (*h*-BN) consists of sp^2^-bonded honeycomb-structured monolayers. Adjacent monolayers have a fixed spacing determined by the interlayer Van der Waals forces^22^. More importantly, the *h*-BN has excellent thermal stability and chemical inertness. All these attributes could be advantageous to serve as GBM for granular L1_0_-FePt thin films. Here, we present a systematic experimental investigation of the fabrication of such FePt-(*h*-BN) granular thin film. X-ray diffraction (XRD), transmission electron microscopy (TEM) analysis, and magnetic hysteresis measurements have been performed to evaluate the microstructure and properties. Concerns over lateral thermal conductivity, which is important for HAMR recording performance, will be addressed at the end of this paper.

## Results and discussion

**Figure 1:**
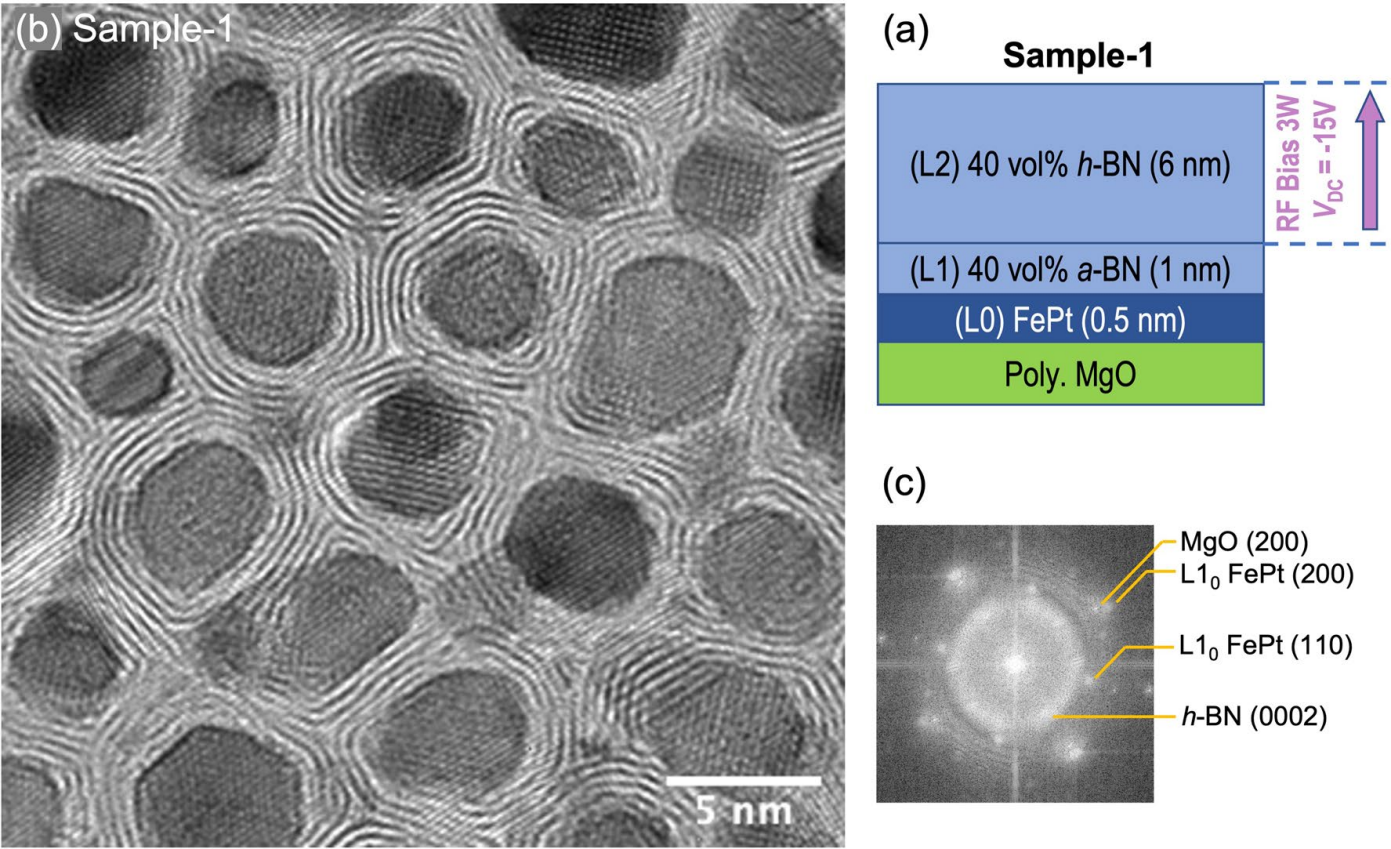
TEM micrograph showing the formation of *h*-BN nanosheets in the grain boundary regions of the FePt-(*h*-BN) nanogranular film. (**a**) Deposition film stack of Sample-1; (**b**) Plane-view HRTEM of Sample-1 and (**c**) FFT pattern.

Figure 1b shows the plane-view high-resolution TEM image of a 7 nm-thick FePt-(*h*-BN) sample with BN concentration of 38vol%, which is referred to as Sample-1. As illustrated in Fig. 1a, Sample-1 was fabricated in the following steps: (L0) a 0.5 nm thick FePt was initially sputtered onto an 8 nm thick MgO underlayer; (L1) an approximate 1 nm thick FePt-BN layer was then deposited using the co-sputtering technique with separate Fe_0.55_Pt_0.45_ and amorphous boron-nitride targets without bias on the substrate; (L2) continued co-sputtering of FePt and BN with a 3W RF bias applied on the sample substrate (*V*_DC_ = -15 V). From Step (L0) to Step (L2), the substrate was heated and maintained at 650 °C within a total deposition time of 6 min.

Sample-1 was fabricated with a volume fraction of BN much higher than the normal for the purpose of better characterization of the grain boundary phase. The image in Fig. 1b shows that the FePt grains are well separated by crystalline boron nitride monolayers that conform to grain boundary surfaces and encircle each grain. The FFT (fast Fourier transform) analysis of this image in Fig. 1c reveals a diffraction ring over the range of 0.33–0.35 nm, matching the *d*-spacing of *h*-BN (0002) planes (*d* = 0.333 nm). In addition, the presence of the FePt {110} superlattice spots shown in the FFT pattern demonstrates the L1_0_ ordering and the [002] texture of FePt grains in this film.

**Figure 2:**
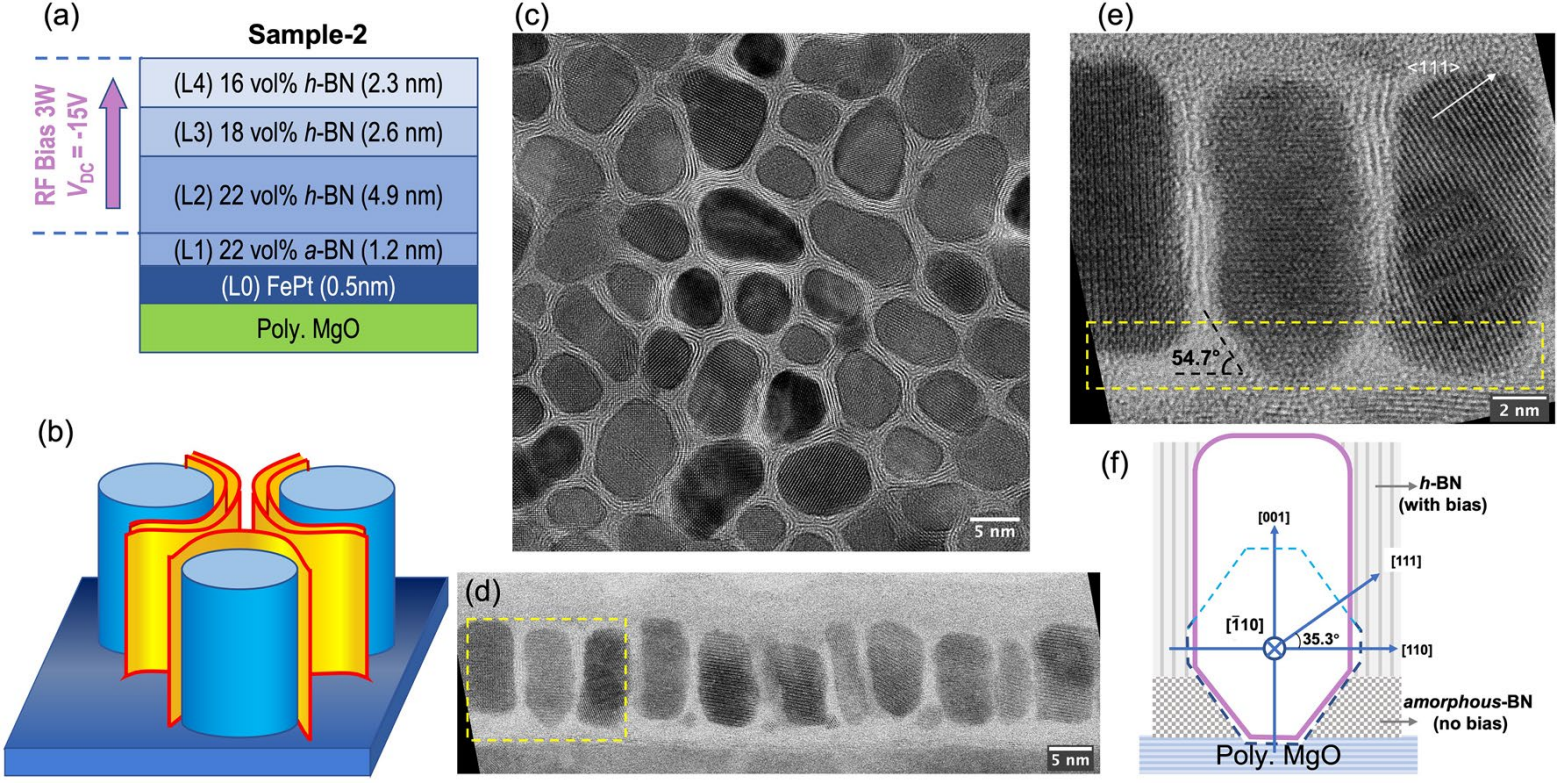
(**a**) Deposition film stack of Sample-2; (**b**) The schematic of the core–shell nanostructure. (**c**–**f**) TEM micrograph analysis of the FePt-(*h*-BN) columnar core–shell structure observed in Sample-2: (**c**) HRTEM plane-view image; (**d**,**e**) STEM-BF cross-sectional image and a magnified high-resolution view of the grain boundaries; (**f**) The schematic demonstrates the viewing axis $[\overline{1 }10]$, the grain shape, and the structure of the BN grain boundary materials. The blue dashed line delineates the Wulff shape of L1_0_ FePt grains at a high temperature viewed from the $[\overline{1 }10]$ direction.

In light of this observation, we further develop the fabrication method for taller FePt grains. In Sample-2, the well-defined granular microstructure was achieved with a much lower BN volume fraction. The lower BN concentration results in the grain boundary width of about 1.5 nm, a value that is normal in most reported granular FePt-X films. Figure 2a shows the film stack of Sample-2, which was fabricated using a procedure similar to Sample-1. The deposition Step (L2-L4) of Sample-2 is refined as shown in Fig. 2a, where the boron nitride concentration was varied through the film thickness with an average volume fraction of 19%. Again, over the entire FePt/FePt-BN deposition process, the substrate temperature was maintained at 650℃. The plane-view HRTEM image of this sample is shown in Fig. 2c. Within the narrow grain boundaries, the parallel *h*-BN nanosheets, with the characteristic interlayer spacing of the ideal *h*-BN phase, can also be observed.

Sample-2’s cross-sectional view in Fig. 2d and a magnified region in Fig. 2e reveal more details of the crystalline nanostructure for both FePt grains and boron nitride grain boundaries. The application of substrate bias is carefully delayed at the start of the FePt-BN co-sputtering, i.e., during Step (L1). In the film grown prior to the application of substrate bias, the BN materials in grain boundaries appear amorphous, and the FePt grains are shown to grow in both lateral and perpendicular directions with specific crystalline facets that match the equilibrium shape (Wulff construction) of the L1_0_ FePt nanocrystal: a truncated octahedron made up of eight {111} planes and six {100} planes^23–25^. The illustration in Fig. 2f shows the view of the Wulff polyhedron along $[\overline{1 }10]$ direction (blue dashed line) matching the bottom of FePt grains, in agreement with the TEM image above. The faceted growth in this step consequently leads to the increase of the lateral sizes of the FePt grain while its height increases. After applying the substrate bias, *h*-BN nanosheets start forming in the grain boundaries, with the honeycomb monolayer growing parallel to the side surfaces of the FePt grains. The initiation of perpendicular *h*-BN nanosheet formation aligns well with the start of columnar growth of the FePt grains. The *h*-BN effectively inhibits the lateral growth of the FePt grains, as the *h*-BN monolayers grow continuously in the boundary regions, keeping up with the growth of FePt grains. Figure 2b presents an illustration of this nanogranular FePt-(*h*-BN) core–shell structure. The constant width of the grain boundary throughout the film thickness is clearly shown in the TEM image in Fig. 2e, as the number of h-BN monolayers in the grain boundaries remains unchanged.

Through a large number of experimental trials, we have established a strong correlation between the application of substrate bias at sufficiently high substrate temperatures and the formation of *h*-BN layers conforming to FePt grain boundaries. Since the amorphous BN portion deposited prior to the application of substrate bias remains amorphous throughout the entire film deposition process, we can conclude that the *h*-BN directly grow at the growing surface. There is no evidence showing that *h*-BN is formed by phase transformation from the amorphous. Furthermore, the initial FePt-(*a*-BN) layer, deposited without substrate bias, results in faceted FePt grain growth that leads to grain size increase with film thickness. Therefore, adjusting the delay of the bias application would enable the control of grain size provided the density of FePt grain nucleation can also be adjusted accordingly to maintain the grain pitch in proportion.

More importantly, we hypothesize that the perpendicular *h*-BN grain boundaries and FePt grains grow simultaneously at the growing surface of the film during deposition, where the *h*-BN honeycomb nanosheets grow along the side surfaces of FePt grains. This hypothesis requires that the FePt grains maintain a height slightly greater than the growing surface of the grain boundaries throughout the deposition process, thereby enabling the growing side surfaces of FePt to guide the growth of h-BN nanosheets. This hypothesis will be discussed later in Fig. 6. After all, matching the growth rates of FePt grains and *h*-BN grain boundaries is important, as their relative height difference needs to be maintained at any instant during the growth. If the growing surfaces of FePt grains are significantly higher than that of the *h*-BN, adjacent FePt grains may grow laterally to connect over the *h*-BN grain boundary in between, which will be discussed later in Fig. 5.

**Figure 3:**
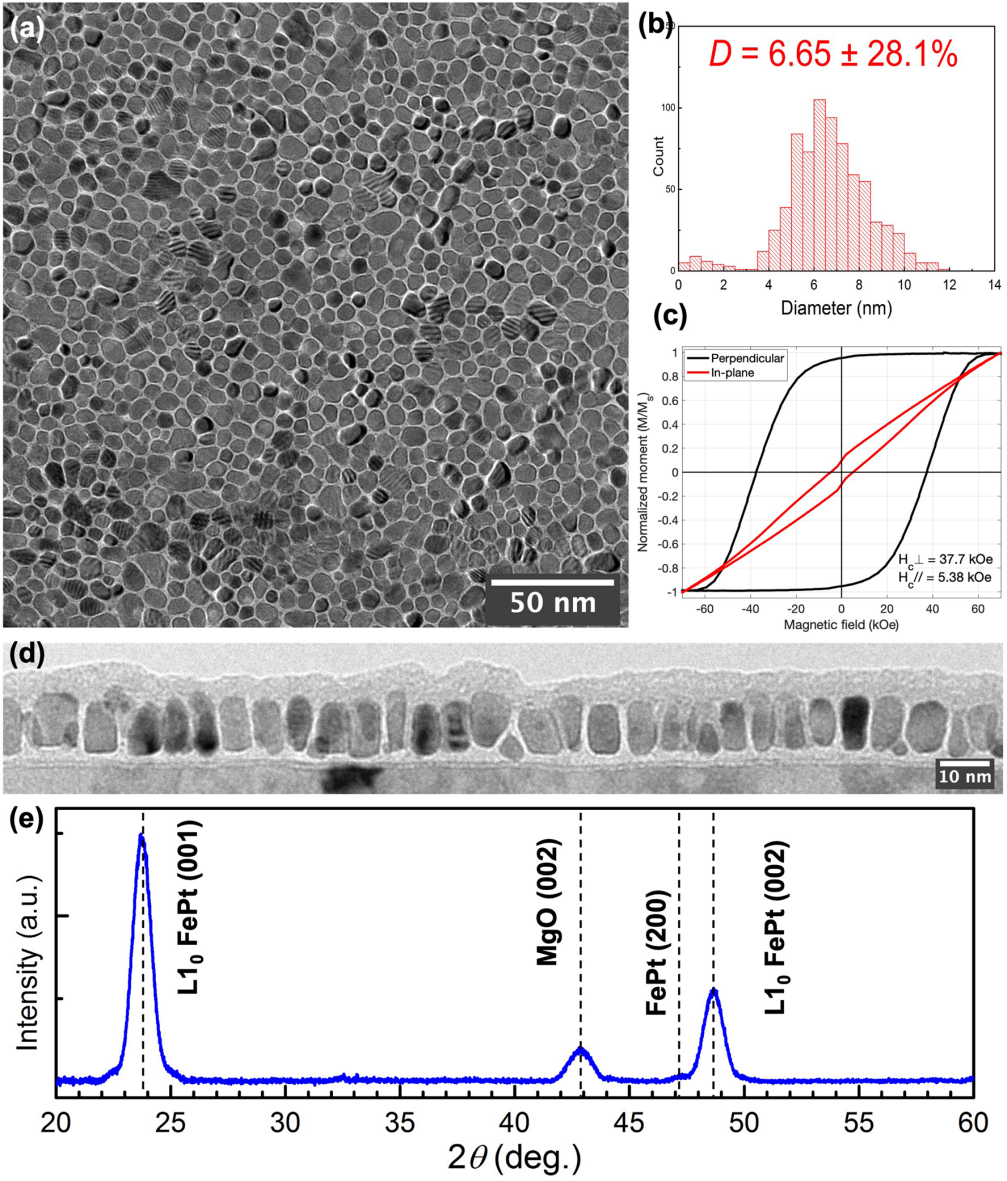
Characterizations of Sample-2 (FePt-19vol% *h*-BN, *t* = 11.5 nm). (**a**) BF-TEM plane-view image; (**b**) grain size distribution; (**c**) magnetic hysteresis loops; (**d**) BF-TEM cross-sectional image; (**e**) Out-of-plane XRD spectra.

The plane-view TEM image over a larger area of Sample-2 is shown in Fig. 3a. The well-defined granular microstructure shows that the majority of FePt grains are completely encircled by the BN grain boundaries, with sparsely distributed exceptions where some adjacent FePt grains are laterally connected. The grain size distribution in Fig. 3b shows the grain size of 6.65 ± 1.87 nm and the grain pitch distance of 8.24 ± 1.93 nm. Consequently, the average grain boundary width is around 1.5 nm, so the number of *h*-BN monolayers within a grain boundary is estimated to be around four, which matches what is shown in cross-section TEM images. Figure 3d shows a typical cross-sectional TEM image of Sample-2. Most grains are columnar, with an average grain height of 11.5 nm. Note that even when some grains are tilted from the film's normal direction, the grain boundary gap width remains the same through the thickness, with the side surfaces of the two adjacent tilted grains growing in parallel. Though most FePt grains have developed tall and columnar shapes with an aspect ratio *h/D* = 1.73, imperfect grains can be clearly seen, which will be characterized later in the paper.

The formation of *h*-BN is the key factor for achieving good granular microstructures. Since the film is formed at relatively high substrate temperatures during the deposition, good atomic ordering of the FePt L1_0_ phase can be readily achieved. Figure 3e shows the out-of-plane X-ray diffraction (XRD) pattern of Sample-2. No (111) peaks are observed, indicating good [001] texture for FePt grains. The integrated intensity ratio of the L1_0_-ordered superlattice peak (001) to the fundamental peak (002) (*I*_*001*_*/I*_*002*_) is about 2.53, and the calibrated order parameter^26^ is *S* = 0.78. A barely visible FePt (200) peak located at the left shoulder of the relatively sharp L1_0_ (002) peak indicates a very small percentage of in-plane oriented grains. The FWHMs of L1_0_ (001) and (002) peaks in the measured rocking curves are around 9.5°. Figure 3c shows the perpendicular and in-plane magnetic hysteresis loops, measured at room temperature, with a perpendicular coercivity of 37.7 kOe and an in-plane coercivity of 5.38 kOe. The opening of the in-plane curve likely results from the combination of in-plane ordered L1_0_ FePt grains and the relatively broad dispersion of FePt c-axis orientation.

**Figure 4:**
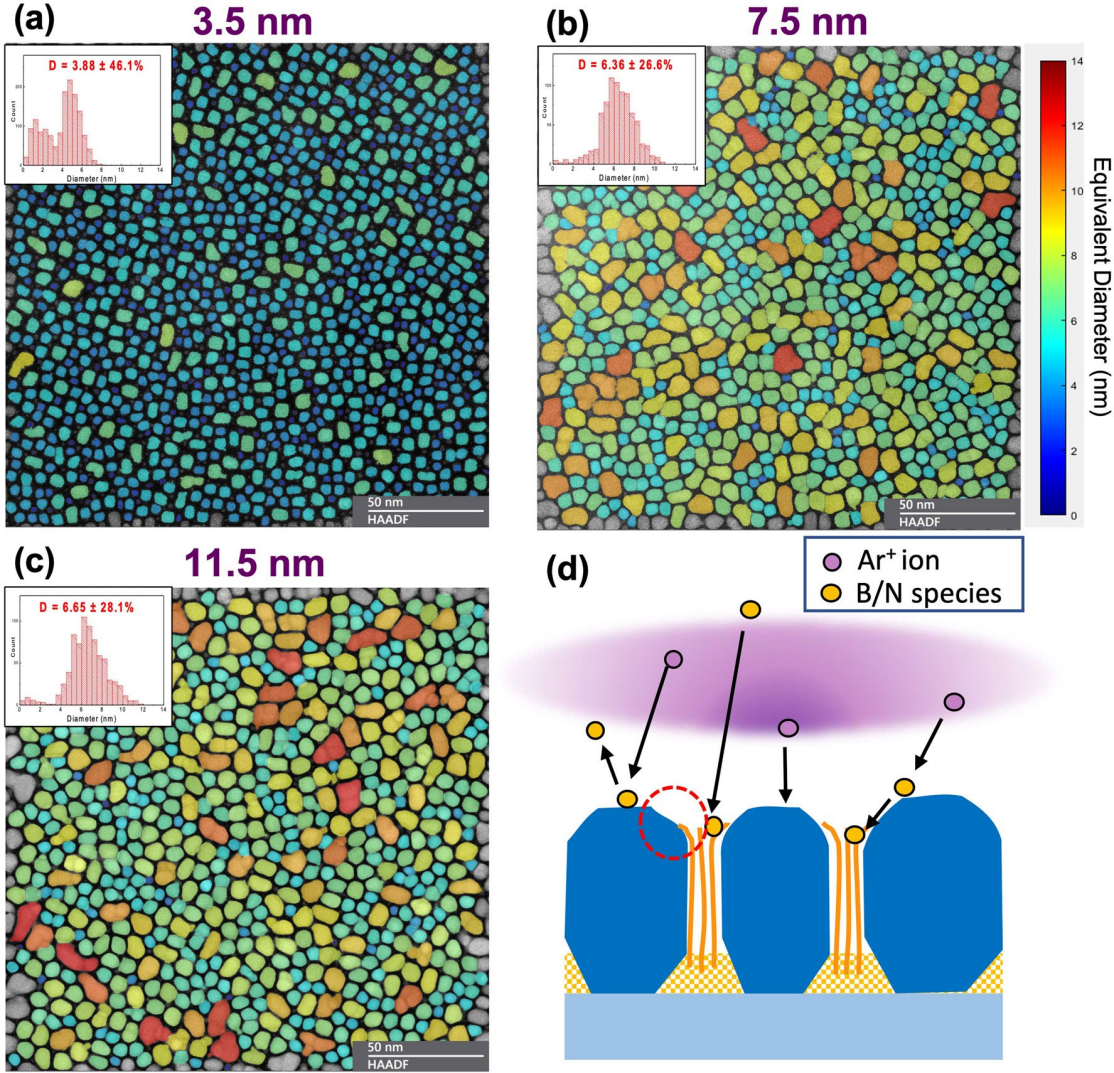
Growth process analysis of the FePt-(*h*-BN) granular film. The false-color STEM-HAADF plane-view images of (**a**) Sample-2α (*t* = 3.5 nm), (**b**) Sample-2β (*t* = 7.5 nm), and (**c**) Sample-2 (*t* = 11.5 nm) share the 50-nm scale bar. (**d**) The diagram shows the hypothesized effect of RF bias: resputtering effect for kicking off weakly bound atoms with *h*-BN monolayers as the surviving phase. As pointed out in the red circle, the *h*-BN grain boundaries are slightly lower than the growth surfaces of FePt grains. The growth of the *h*-BN nanosheets may be guided by the side surfaces of FePt.

Plane-view STEM images in high-angle annular dark field mode (HAADF) have been taken for three FePt-BN samples, with the deposition processes stopped at different stages for Sample-2’s film stack shown in Fig. 2a. Figures 4a–c show the images for these samples with different thicknesses, referred to as Sample-2α (*t* = 3.5 nm), Sample-2β (*t* = 7.5 nm) and Sample-2 (*t* = 11.5 nm), respectively. The false color in the images represents the lateral size of FePt grains corresponding to the unified color map. The image shown in Fig. 4a, Sample-2α, shows the plane-view of FePt grains mainly with *a*-BN grain boundaries that are deposited without substrate bias. Most grains have square or rectangular shapes with edges parallel to < 110 > directions (with high-resolution images presented in Supplementary Fig. S2(a)), which agrees with the top-down view (along [001] direction) of the Wulff polyhedron of FePt and is consistent with the faceted growth analysis provided earlier. There also exists a significant percentage of very small grains as the bimodal distribution of grain sizes is evident at this early growth stage. At the film thickness of 7.5 nm, as shown in the image of Fig. 4b, not only did the lateral grain size become larger compared to Sample-2α, but also the bimodal distribution shown in Fig. 4a has evolved to a single peak with the disappearance of the smaller-grain-size peak. Since the substrate bias had already been started by the moment of 3.5 nm film thickness, *h*-BN should have been forming in grain boundaries during the film growth between Sample-2α and Sample-2β. Therefore, it is reasonable to think that adjacent grains with grain boundary separation less than the space needed to fit in at least one *h*-BN monolayer are likely to merge, especially when the two grains are nucleated on the same MgO grain of the underlayer. This is one possible mechanism for the disappearance of the bimodal distribution. We will discuss other possible mechanisms later.

**Figure 5:**
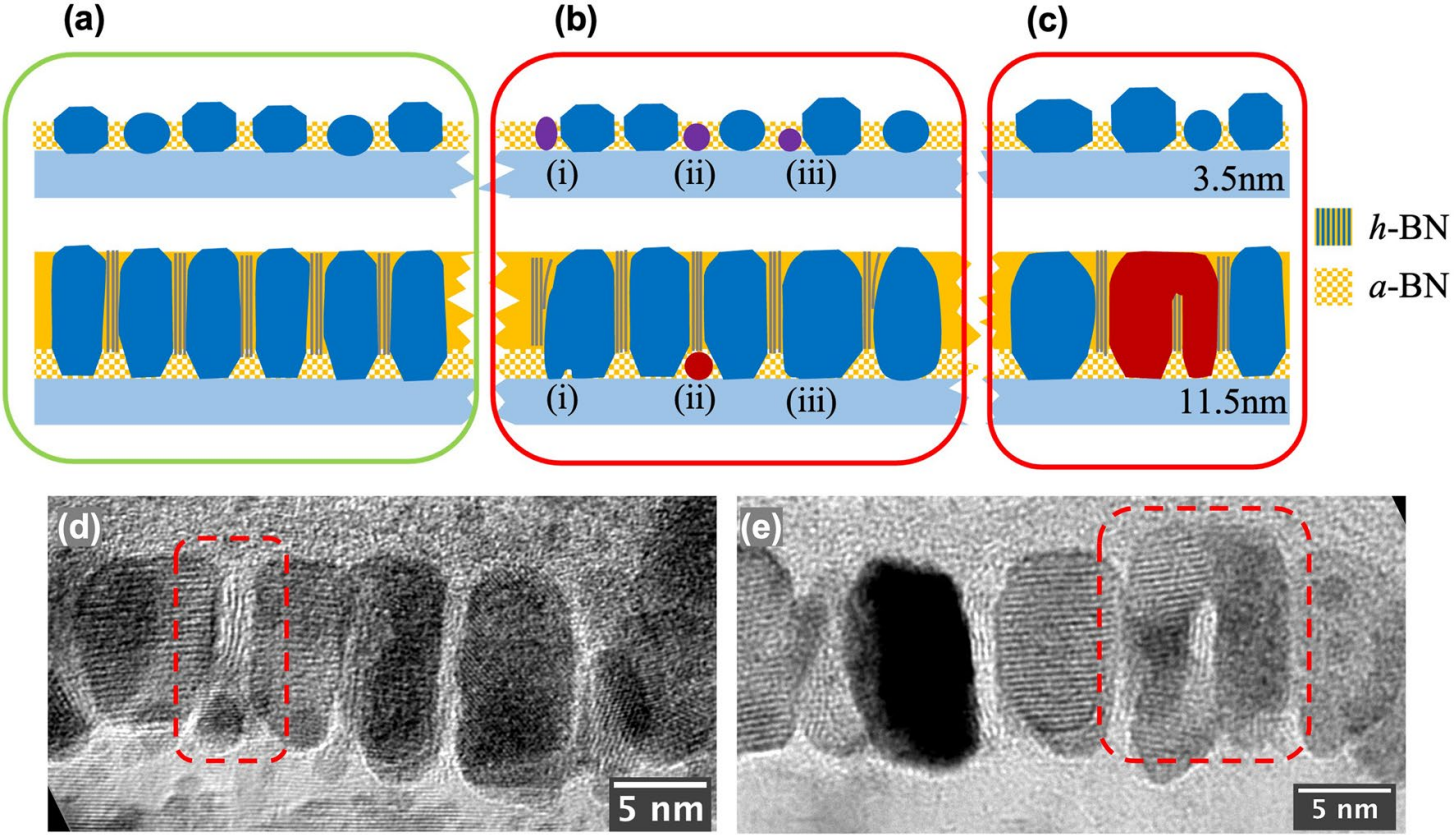
Characterization of L1_0_ FePt granular film with boron nitride grain boundaries. (**a**) Illustration of columnar growth of most FePt grains: In the FePt-(*a*-BN) layer, FePt grains exhibit facet growth, with their lateral size increasing with film thickness. With RF-bias turned on, *h*-BN starts to form inside grain boundaries, facilitating the columnar growth of FePt grains. (**b**) Illustrations of three types of defect growth: (i) a small grain closely placed to a large one gradually grow together to form a larger grain; (ii) A small FePt grain shadowed by two adjacent FePt grains allowing the* h*-BN grain boundary to grow on top; (iii) two small grains coalesce in the early stage of film growth, resulting in a much larger size grain. (**c**) Lateral top-connection of adjacent FePt grains shielding the growth of *h*-BN grain boundary. (**d**) Cross-sectional HRTEM image showing clear evidence for the defective growth illustrated in (**b**); (**e**) Image showing the evidence for the top-connected adjacent FePt grains illustrated in (**c**).

Comparing the two images shown in Fig. 4b and c, we learn that the grain size distribution is basically unchanged as the film grows from 7.5 nm to 11.5 nm in thickness. This is consistent with the fact that FePt grains grow largely columnar as facilitated by the *h*-BN monolayers in the grain boundaries. However, in both images, laterally connected FePt grains can be seen, whereas they are not present at the 3.5 nm case (Fig. 4a). These connected grains will be discussed in detail when we discuss Fig. 5. Figure 4d illustrates our proposed mechanism of how the application of substrate bias facilitates the formation of *h*-BN in the grain boundary. We believe that the resputtering caused by substrate bias is an important factor. Without substrate bias, deposited BN in the grain boundary is amorphous. The applied RF bias induces energetic ion bombardments that preferentially knock off weakly bonded B/N species, whereas strongly bonded BN can survive. Thus, the *h*-BN phase is much more likely to survive due to the strong covalent bonds between boron and nitrogen atoms within the (0002) hexagonal monolayers.

The illustration in Fig. 5a provides a detailed characterization of normal columnar growth for about 90% of the grains; Fig. 5b and c illustrate two major types of defective growth for the other 10% of the grains. The 3.5 nm thick case (upper half parts) reflects the bimodal nucleation. Figure 5b illustrates: (i) how a small grain nucleated adjacent to a much larger grain can grow towards the larger grain and eventually merges with it to become a single grain; (ii) a small grain nucleated in between two relatively larger grains can be shadowed during the film growth, providing the chance for boron nitride material to grow over the grain, a mechanism for forming a wider *h*-BN grain boundary; (iii) lateral coalesce of a large grain with a small grain close by in the early stage of the growth. Note that since the MgO grain size of the underlayer is much larger than that of FePt grains, the smaller and larger grains in both cases (i) and (iii) are more likely to be on top of the same MgO grain, in which case the lattices of the two FePt grains would match to form a single crystal with smaller free energy. Figure 5c illustrates a different kind of defective growth that happens at a relatively later stage of film growth. If an *h*-BN grain boundary grows slower than that of the two FePt grains at either side, the two grains can grow laterally, starting to shield the arrival of the B/N species and eventually closing up the grain boundary gap from both sides. Several top-connected grains can be seen in the TEM image shown in Fig. 4c, though they are sparsely distributed. High-resolution cross-sectional TEM images in Fig. 5d, e and Supplementary Figs. S2(d)-(e) show clear evidence for the illustrations in Fig. 5a–c and the accompanied discussion.

**Figure 6:**
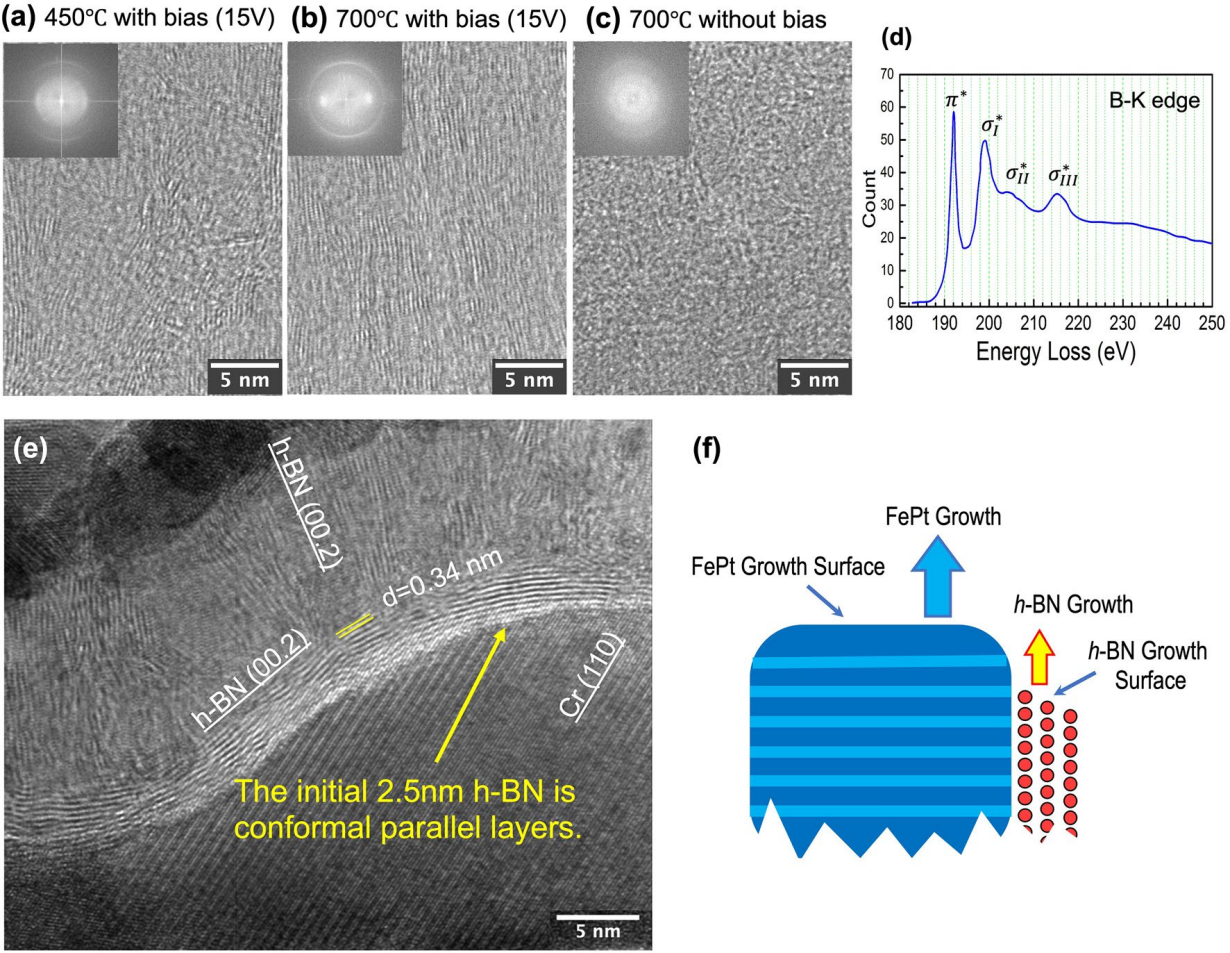
(**a–c**) Cross-sectional HRTEM images and corresponding FFT patterns of three BN films deposited with conditions: (**a**) 450 °C with RF bias; (**b**) 700 °C with RF bias; (**c**) 700 °C without applying bias. (**d**) Electron energy loss spectrum (EELS) acquired on *h*-BN regions, showing the B-K edge with one ${\pi }^{*}$ peak (191.8 eV) and three ${\sigma }^{*}$ peaks (198.8 eV, 204.2 eV, 215.2 eV). (**e**) Cross-sectional HRTEM image of the 13-nm *h*-BN blanket film on the curved Cr surface. The *h*-BN was RF-sputtered at 650℃, with a 3W RF-substrate-bias. (**f**) The illustration of the proposed growth mechanism of *h*-BN nanosheets at the growth surfaces of the FePt-(*h*-BN) granular films.

Three blanket BN films were deposited with different conditions to investigate the critical conditions for *h*-BN formation. The control sample shown in Fig. 6b was deposited at 700 °C with a substrate bias of 3W to yield the *h*-BN phase, as verified by its FFT pattern. In comparison, when removing the bias (Fig. 6c) the resulting BN film was completely amorphous. Alternatively, when the temperature was lowered to 450 °C while the bias was maintained, layered structures were observed in Fig. 6a, but they were poorly crystallized. These three results reveal that substrate bias is a necessary condition for the formation of *h*-BN, while high temperature improves the crystallinity of the film. The electron energy loss spectrum (EELS), Fig. 6d, of the *h*-BN sample shows four peaks in the Boron-K edge: one ${\pi }^{*}$ peak at 191.8 eV and three ${\sigma }^{*}$ peaks at 198.8 eV, 204.2 eV, 215.2 eV respectively, matching the characteristic peaks of the standard *h*-BN^27,28^. The strong ${\pi }^{*}$ peak confirms the existence of *h*-BN nanosheets with sp^2^-hybridized covalent bonds.

To understand the growth of *h*-BN grain boundaries and the orientation of the nanosheets, the following modeled experiment was carried out. In this experiment, a 13-nm-thick boron nitride film was sputtered on a curved Cr surface with the substrate heated to 650℃ and an RF bias power of 3W applied (*V*_DC_ = -15 V), the same conditions used for producing Sample-1 shown in Fig. 1. Figure 6e shows a cross-sectional HRTEM image of the film where the lattice fringes of *h*-BN monolayers can be clearly observed. The initial 6–7 monolayers of *h*-BN grow parallel to the curved Cr surface, regardless of the curvature of the surface. Namely, on either convex or concave surface locations, the *h*-BN monolayers are all conformal to the metal surface.

With the same two key sputtering conditions, i.e., the substrate bias and the elevated substrate temperature used in the FePt-(*h*-BN) film growth with co-sputtering of FePt and BN, the deposition of BN on a Cr surface yields direct growth of *h*-BN with monolayers conforming to the surface regardless of its curvature. We also do not observe any epitaxial growth at the *h*-BN/Cr interfaces, so this phenomenon is unrelated to the crystalline structures and lattice orientations of Cr. These observations support that the surface conformal growth of *h*-BN are indeed an interface-related phenomenon. Then, this result offers a direct insight into the growth of *h*-BN grain boundaries in the granular FePt films, as illustrated in Figs. 1–4. During the co-sputtering of FePt and BN under the two key sputtering conditions, if the top growth surface of an FePt grain is always slightly higher than the top growth surface of the grain boundary on the side, this would leave an uncovered perpendicular surface of the FePt grain "exposed". The *h*-BN nanosheets would then follow the exposed FePt side surfaces and extend its hexagonal monolayers along, as demonstrated in Fig. 6f. This picture of the *h*-BN grain boundary growth matches exactly with the observations in the modeled experiment results of the bilayer structures, despite that the granular FePt-(*h*-BN) system goes through an additional step of phase separation. By analyzing a large number of cross-sectional TEM images, we do find that the heights of FePt grains are always slightly higher with the *h*-BN grain boundaries, consistent with the above analysis.

Although in this modeled experiment, Cr, instead of FePt, is used, a follow-on experiment using FePt as underlayer shows similar results with *h*-BN nanosheets directly forming on FePt surface with monolayers conforming to the surface. However, this phenomenon does not occur on a pure Pt surface with the same sputter conditions, indicating direct *h*-BN growth may also depend on certain surface properties of the metal.

In addition, the growth of *h*-BN monolayers conforming to transition metal surfaces has been commonly observed^29,30^, using either chemical vapor deposition (CVD) or physical vapor deposition (PVD) techniques. The low surface energy of *h*-BN monolayer^31^ and various interface interactions^29^ have been investigated as the contributing factors to this phenomenon. For example, previous studies have found that for *h*-BN honeycomb monolayer on various metal surfaces^32^, the electronic structure of the *h*-BN monolayer remains largely unchanged due to strong intralayer bonding. It is also known that *h*-BN monolayers exhibit localized charge centers^22,32^ (these charge centers are responsible for shifted matching for multilayer stacking and give rise to the Van der Waals interactions between the layers). The conductive metal surface could behave as a charge “mirror”, creating imaging charges of the charge centers in the monolayer, creating attractive forces causing the monolayer basal planes to be parallel to the metal surface.

## Summary and remarks

A systematic experimental study was conducted to fabricate granular L1_0_-FePt thin film media with boron nitride GBM. The film media were deposited using co-sputtering technique with separate targets of FePt and *a*-BN. Heating the substrate above 650 °C, the initial deposition creates FePt grains with faceted growth surrounded with *a*-BN grain boundaries, and FePt grain size increases with increasing film thickness. At an appropriate point, an RF bias was applied to substrate. The substrate RF-bias yields the formation of *h*-BN nanosheets inside grain boundaries with their monolayers parallel to the side surfaces of FePt grains. The number of *h*-BN monolayers mostly remained constant in a grain boundary throughout the thickness, facilitating columnar growth for most of the FePt grains in the film sample. The study shown here indicates that it is possible to grow very tall FePt grains without increasing their lateral size while keeping good granular microstructure, as long as the growth rate of FePt grains and the growth rate of *h*-BN inside grain boundaries can be matched constantly during film deposition. The deposition-rate matching should prevent the lateral connection between adjacent FePt grains.

For HAMR application, the thermal conductivity between adjacent FePt grains is naturally an important concern. Before any discussion, we need to note that the thermal conductivity in *h*-BN is highly anisotropic: the lateral thermal conductivity within each honeycomb monolayer is relatively high^33^ (${\kappa }_{//}$> 200 W/(m·K)), and the thermal conductivity perpendicular to the plane of the honeycomb monolayer is two orders of magnitude lower^34^ (${\kappa }_{\perp }\approx$ 2 W/(m·K)). For the granular FePt-(*h*-BN) film fabricated here, thermal conductivity is likely to be dominated by the perpendicular thermal conductivity though it needs to be confirmed experimentally because of possible thermal conduction tangent to FePt grain boundaries. Nevertheless, experimental characterization of lateral thermal conductivity for the media achieved here is very much needed in future investigations.

## Methods

### Sample preparation

In this study, all thin film samples were deposited on Si (001) substrates using an AJA sputtering system with base pressures of 2 × 10^–8^ Torr or less. The underlayer consisted of a 4-nm amorphous Ta adhesion layer and a 30-nm Cr with a (002) texture to produce large grains and enhance the texture of subsequent films. As the seed layer for L1_0_-FePt grains to form the chemical ordering and c-axis normal to the film plane, 8 nm of MgO was epitaxially deposited on Cr. The underlayer's growth parameters are selected to achieve a low grain boundary density, low surface roughness, and a strong MgO [002] texture (rocking curve ≤ 8°).

The magnetic layers are composed of L1_0_ FePt grains and oriented *h*-BN nanosheets, deposited by co-sputtering Fe_0.55_ Pt_0.45_ and *a*-BN targets at 650 °C and 5 mTorr of Ar in the chamber. The distance from substrate to targets was roughly 20 cm. A 3 ~ 4W RF bias was applied to the substrates of certain samples and the substrate direct-current voltage (*V*_DC_) at steady state was around -15V. The volume fraction and thickness were estimated with the deposition rates of different materials, which were pre-calibrated separately using transmission electron microscopy (TEM) imaging. The deposition rate of *h*-BN is quite low (~ 0.007 nm/s with 180W). All samples' recording layers were fabricated in a multi-step way, similar to what is illustrated in Fig. 2a. The initial layer (designated as [L0] layer) was a 0.5-nm pure FePt nucleation layer. Then the co-sputtered FePt-BN layers always began with a [L1] layer deposited without substrate bias, followed by a [L2] layer containing the same volume fraction of BN with bias. It was discovered that when the substrate bias was applied to the entire magnetic layer, the resulting microstructure and ordering were poor. This delayed bias was intended to prevent the disruption of the nucleation stage and was shown to improve the final microstructure. For samples of different thicknesses, one to three sublayers with slightly lower BN volume fractions were then deposited. Essentially, the BN vol % decreases gradually from the bottom sublayer to the top. It has been demonstrated that this stack with graded volume fractions prevents GBM from capping the top of FePt grains, thereby inhibiting the formation of second layers in the FePt-SiO_2_ system. It turns out to be effective in the FePt-BN system as well. We denote the sample with 38vol% BN and thickness of 7.5 nm as Sample-1, whose film stack is FePt (0.5 nm) / FePt-40vol% *a*-BN (1 nm) / FePt-40vol% *h*-BN (6 nm). We denote the sample with 19vol% BN and thickness of 11.5 nm as Sample-2, whose film stack is FePt (0.5 nm) / FePt-22vol% *a*-BN (1.2 nm) / FePt-22vol% *h*-BN (4.9 nm) / FePt-18vol% *h*-BN (2.6 nm) / FePt-16vol% *h*-BN (2.3 nm). The film stacks of them are also shown in Figs. 1a and 2a.

### Sample characterizations

The degree of chemical ordering of FePt and crystalline texture of all the layers were examined using the traditional X-ray diffraction (XRD) technique with Cu Kα radiation. The superconducting quantum interface device vibrating sample magnetometer (SQUID-VSM) (Quantum Design MPMS3 system) with an applied magnetic field up to 7 T was used to measure the magnetic properties at room temperature. The plane-view and cross-sectional transmission electron microscopy (TEM) imaging was used to assess the microstructure of the samples, including bright-field TEM (BF-TEM), high-resolution TEM (HR-TEM), scanning TEM-high angle annular dark field and bright field (STEM-HAADF & STEM-BF) techniques. For accuracy and consistency, the STEM-HAADF plan-view images and image processing software MIPAR were used to analyze the grain size and grain center-to-center pitch distances. For each grain, the pitch distance analysis counted its six closest neighbors. The in plane- STEM-HADDF images of the grains are colored based on the equivalent grain diameters.

## Supplementary Information

Supplementary Information.

## Data Availability

All data gathered and/or analyzed in this study are included in the main article and its Supplementary Information files.
