# Supplementary material for: Bias sputtering of granular L10-FePt films with hexagonal boron nitride grain boundaries
Source: Sci Rep. 2023 Jul 8;13:11087. doi: 10.1038/s41598-023-38106-9 (PMC10329668; doi:10.1038/s41598-023-38106-9)
Supplement: Supplementary file 1 — Supplementary Information. [file 41598_2023_38106_MOESM1_ESM.pdf]

Supplementary Information

Bias sputtering of granular $L1_0$-FePt film with hexagonal boron nitride grain boundaries

Chengchao Xu[1, 2, *], B.S.D.Ch.S Varaprasad[1, 2], David E. Laughlin[1, 2, 3], and Jian-Gang (Jimmy) Zhu[1, 2, 3].

1) Data Storage Systems Center, Carnegie Mellon University, Pittsburgh, Pennsylvania 15213, USA

2) Electrical and Computer Engineering Department, Carnegie Mellon University, Pittsburgh, Pennsylvania 15213, USA

3) Materials Science and Engineering Department, Carnegie Mellon University, Pittsburgh, Pennsylvania 15213, USA

* Correspondence and requests for materials should be addressed to C.X. (email: chengchx@andrew.cmu.edu)

1. Resputter effect of substrate bias

We believe that the resputter effect caused by substrate bias is an important factor for the formation of *h*-BN phase. Without substrate bias, deposited BN in the grain boundary is amorphous. The applied RF bias induces energetic ion bombardments that preferentially knock off weakly bonded B/N species, whereas strongly bonded BN can survive. Thus, the h-BN phase is much more likely to survive due to the strong covalent bonds between boron and nitrogen atoms within the hexagonal monolayers. The hypothesis comes from our measurements of the sputter rate of BN under different biases. We deposited thick, pure BN layers on the FePt underlayer at 700°C and calibrated the deposition rates with cross-sectional TEM images. The rates were found to be highly dependent on the bias voltage, V_{DC}, as shown in Table S1. Interestingly, the sputter rate does not decrease monotonically as the RF bias power increases. Generally, substrate bias reduces the sputter rate because it increases the probability of deflecting BN species, which have a lighter mass, and the ions striking substrate surface even cause some etching effects, i.e., resputtering effect. However, we noticed that, compared to no bias, a 15V bias actually increased the rate, implying the formation of a new phase. We indeed observed the formation of *h*-BN nanosheets in the TEM image shown in Fig. 6(b). The *h*-BN deposited on the substrate surface

was less affected by ion bombardment. However, further increasing the bias voltage led to the domination of the resputtering effect, and *h*-BN also began to be ejected from the substrate. We believe this observation supports the mechanism proposed at the beginning of this paragraph.

Table S1. Sputter rate of BN (150W) under different bias voltages

RF Bias power (W)	0	3	5
Substrate voltage V_{DC} (V)	0	-15	-24
Main component	*a*-BN	*h*-BN	*h*-BN
Sputter rate (nm/min)	0.092	0.197	0.132

2. Growth process analysis of FePt-BN films

To better understand the growth process, Sample-2α (t = 3.5 nm) and Sample-2β (t = 7.5 nm) were fabricated using the same film stack design and deposition conditions as Sample-2 (film stack shown in Fig. 2(a)), except for the thickness. They are intended to represent the three stages of the film growth for Sample-2. The magnetic hysteresis loops and XRD patterns of these samples with varying thicknesses are shown in Figure S1. The high-resolution STEM images of Sample-2α and Sample-2 are presented in Figure S2. As mentioned before, Sample-2α was designed to illustrate the nucleation stage of FePt with *a*-BN grain boundaries deposited without RF bias. In its perpendicular hysteresis loop (Fig. S1(a)), there is a notable soft kink near zero fields, which should be the result of the smaller FePt nuclei (as shown in Fig. S2(a)). The smaller FePt grains with higher surface-to-volume ratios normally show lower anisotropy fields or coercivities, which is also known as the finite size effect. As the film grows thicker, Sample-2β (t = 7.5nm) exhibits a significant improvement of perpendicular coercivity ($H_{C\perp}$) and loop squareness (Fig. S1(b)). This agrees with the change of microstructures (Fig. 4) that the grain size rose dramatically, and the bimodal size distribution disappeared. Only ~8% of the smaller nuclei (D < 3nm) remained during the evolution from Sample-2α to Sample-2β, since they were either shadowed by the adjacent larger grains or coalesced with others to form larger ones. It also contributes to the disappearance of the soft kinks near zero fields. In Sample-2β's out-of-plane XRD pattern (Fig. S1(d)), the peak widths (FWHM) of $L1_0$-FePt (001) and (002) peaks were decreased, and the intensity ratio $I_{(001)}/I_{(002)}$ was higher compared with Sample-2α. Then, during the growth from 7.5nm to 11.5nm, the microstructure and magnetic properties stabilize and change little.

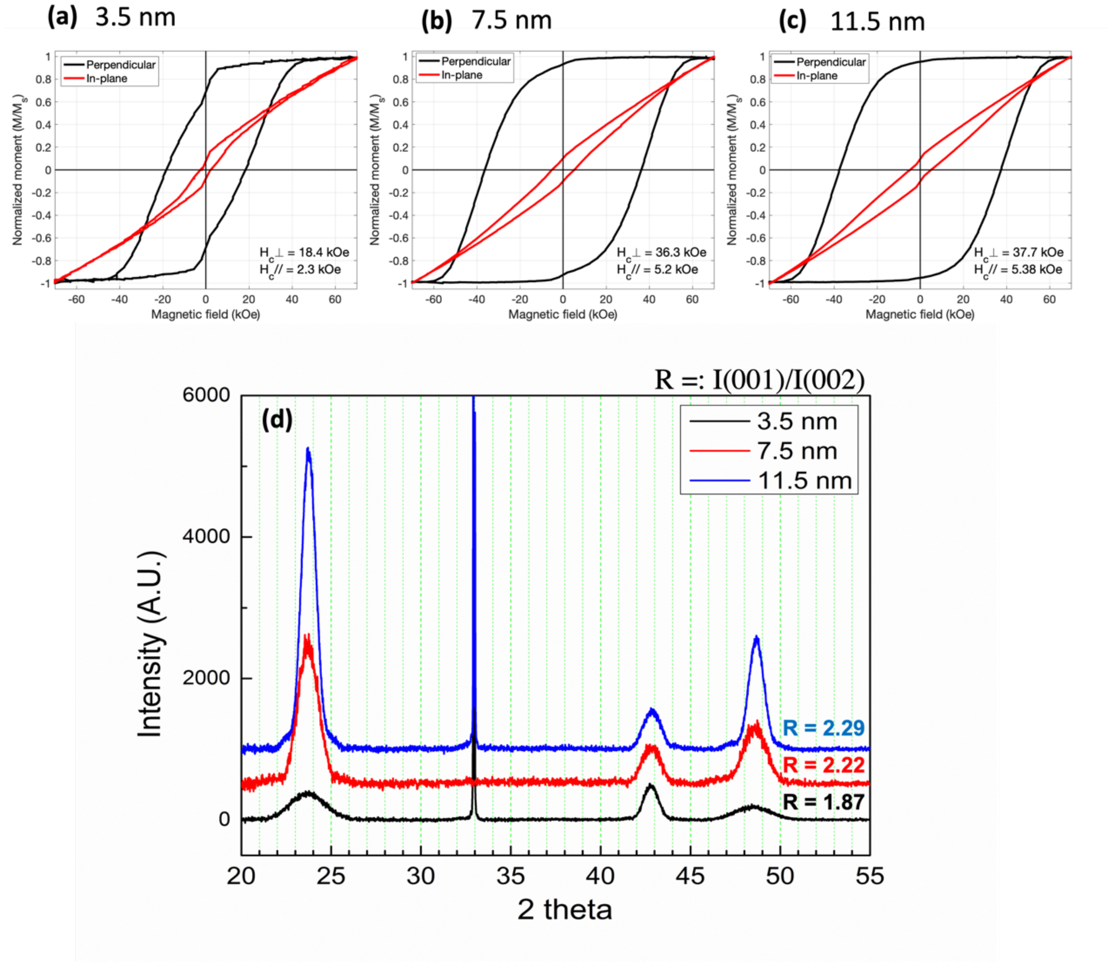

Figure S1. Properties of FePt/BN films with different thicknesses to study the growth processes. (a)-(c) Magnetic hysteresis loops for (a) Sample-2α (*t* = 3.5 nm), (b) Sample-2β (*t* = 7.5 nm), (c) Sample-2 (*t* = 11.5 nm). (d) XRD patterns of the three samples above (*t* = 11.5, 7.5, 3.5nm from top to bottom).

$L1_0$ ordered FePt grains with the desired [001] texture and lateral (projected) grain edges parallel with <110> orientations were obtained in Sample-2α, as shown in Fig. S2(a). The rectangular shapes of nuclei agree with the Wulff polyhedron of $L1_0$ FePt nanocrystals viewed along the [001] direction. Some of the large nuclei in the cross-sectional image, Fig. S2(b), also exhibit {100} and {111} facets. Bimodal size distribution was developed in the nucleation stage.

Figure S2(c-e) are supplementary TEM images for Sample-2(11.5nm, FePt-19vol% *h*-BN). Figure S2(c) shows an atomic resolution STEM-HAADF image of Sample-2, where we can

observe large grains with correct texture and few tiny grains that appear to be buried at the bottom of the film. Figure S2(d) showcases a typical microstructure imperfection, lateral grain connection, which is sparsely located in both Sample-2β and Sample-2. Because of the overall deficit of the *h*-BN grain boundary materials (GBM), lateral grain connections on top of *h*-BN at various heights are possible, eventually forming elongated grains or warm-shaped large grains. Figure S2(e) shows an example of defective growth illustrated in Fig. 5(b).(i) and described in the paper.

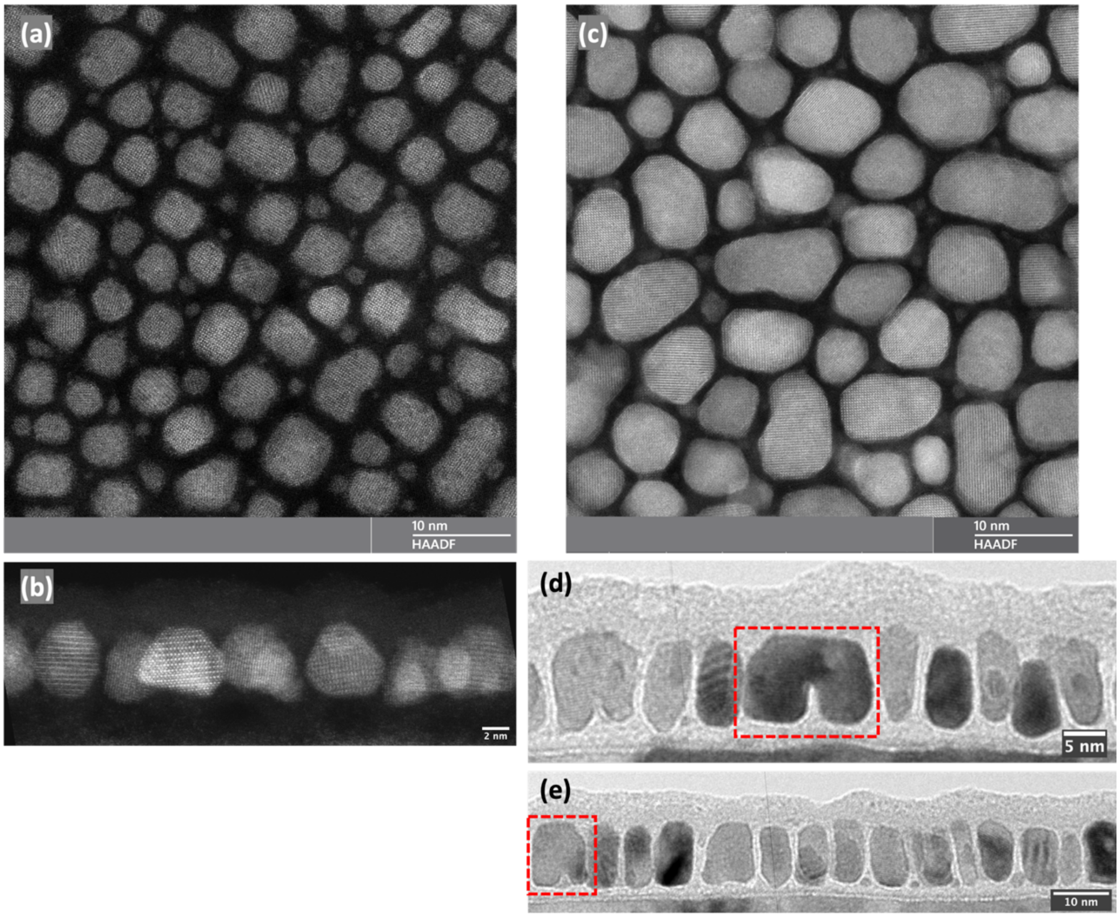

Figure S2. TEM analysis of the microstructure of FePt/BN films with different thicknesses. (Left) The STEM-HADDF plane-view image(a) and cross-sectional image (b) of Sample-2α (t = 3.5 nm). (Right)The STEM-HADDF plane-view image(c) and BF cross-sectional image (d,e) of Sample-2, in the region that defective grains exist as labeled by the red rectangles.

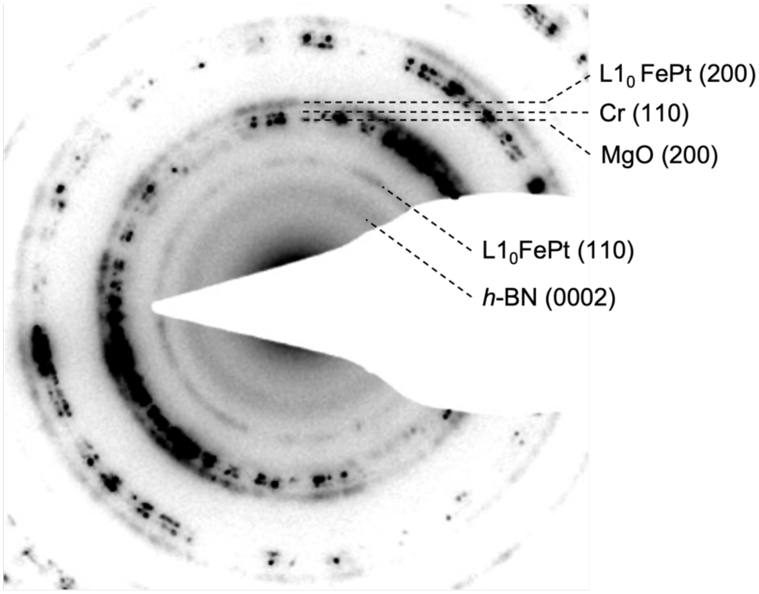

Figure S3. Out-of-plane selected area electron diffraction (SAED) pattern of Sample-2.(electron beam is normal to the film plane). The existence of the *h*-BN (0002) ring corroborates the formation of the *h*-BN phase. The ring appears foggy because the *h*-BN nanosheets are bent to surround the side surfaces of FePt grains, thereby providing excellent grain separation in the granular films.

3. Other potential effects of substrate bias

Notably, we observe in Fig. 6 (d) that away from the substrate surface, the basal planes of *h*-BN gradually turn perpendicular to the substrate surface, i.e., c-axis along the film surfaces. The perpendicular *h*-BN basal planes have been shown to be the energy-favored orientation as the biaxial compressive stress builds up when the film grows thicker[1], and it's a common phenomenon that has been previously reported. Moreover, the substrate bias induces ion bombardments on the growing surface, which can also raise the in-plane compressive stress due to ion peening effect[2,3]. This could be a contributing factor for the perpendicular orientation of *h*-BN layers in the FePt-(*h*-BN) nanogranular structure, which form at high temperatures. However, after the deposited films cool down, we observe no sign of residual compressive stress, but the expansion of h-BN interlayer spacing up to 0.35nm in the grain boundaries (as indicated by the wider ring in the FFT pattern in Fig. 1(c)). C. Androulidakis, et al.[4] find that few-layer *h*-BN exhibits a negative thermal expansion

coefficient. Thus, this observation may stem from the expansion of *h*-BN coupled with the contraction of FePt grains during cooling after deposition. This hypothesis needs further in-situ measurements during the film growth process.

References

1. Kester, D. J., Ailey, K. S. & Davis, R. F. Deposition and characterization of boron nitride thin films. *Diamond and Related Materials* **3.4-6**, 332-336 (1994).
2. Varghese, B. *et al.* Substrate Bias Effects on Magnetic and Structural Properties of L10-FePt Based Recording Media. *IEEE Trans Magn* **52**, (2016).
3. Janssen, G. C. A. M. Stress and strain in polycrystalline thin films. *Thin Solid Films* **515**, 6654–6664 Preprint at https://doi.org/10.1016/j.tsf.2007.03.007 (2007).
4. Androulidakis, C. & Galiotis, C. Thermomechanical behaviour of hexagonal boron nitride at elevated temperatures. *2d Mater* **7**, (2020).
